# Impact of adult growth hormone deficiency on daily functioning and well-being

**DOI:** 10.1186/1756-0500-7-813

**Published:** 2014-11-18

**Authors:** Meryl Brod, Betsy Pohlman, Lise Højbjerre, Johan Erpur Adalsteinsson, Michael Højby Rasmussen

**Affiliations:** The Brod Group, 219 Julia Avenue, Mill Valley, CA 94941 USA; Novo Nordisk A/S, Global Development, Vandtårnsvej 114, DK-2860 Søborg, Denmark

**Keywords:** Growth hormone deficiency, Adults, Patient-reported outcome measure, Daily functioning, Well-being

## Abstract

**Background:**

Adult Growth Hormone Deficiency (AGHD) is a debilitating condition resulting from tumors, pituitary surgery, radiation of the head, head injury, or hypothalamic-pituitary disease. This qualitative study was conducted to better understand the multi-faceted impacts and treatment effects of GHD on adult patients’ daily lives.

Seven focus groups and four telephone interviews were conducted in three countries. Eligible AGHD patients were age 22 or older who had started and stopped growth hormone treatment at least once as an adult. Transcripts were analyzed thematically.

**Results:**

Thirty-nine patients were interviewed; majority etiology was pituitary disease or tumor (62%). Thirty-four patients (87%) were currently on growth hormone replacement therapy; therapy initiation mean age was 43 years. Analysis identified five domains of disease impact: 1) Psychological Health - changed body or self-image and negative emotional impacts; 2) Physical Health - problems with sleep/fatigue, sex drive, weight gain, hair, skin, muscle/bone loss; 3) Cognition - concentration or memory trouble; 4) Energy Loss and its negative impacts (productivity, exercise, chores, socialization, or motivation); and 5) Treatment Effect - treatment enhances quality of life, enabling patients to increase effort (exercise, chores, or work improvements). Energy and sleep are improved. Saturation of themes was reached after the sixth focus group. A conceptual model of GHD disease impacts was developed.

**Conclusions:**

Untreated AGHD has significant negative impacts for patients, which treatment often improves. It is important for clinicians and researchers to understand these multiple impacts so that they can address them in individualized treatment plans and incorporate them when assessing treatment outcomes.

## Background

Adult Growth Hormone Deficiency (AGHD) is a debilitating condition, associated with reduced muscle mass and muscle strength, reduced bone mass or osteoporosis and an increase in body fat [1, 2]. Those with AGHD include adults who were growth hormone deficient as children and become adults with AGHD or adults who become growth hormone deficient due to tumors, pituitary surgery, radiation of the head, head injury or hypothalamic-pituitary disease. AGHD leads to increased morbidity and increased incidence of cardiovascular events, a main cause of the increased mortality observed in this population. A recent review of the epidemiology of growth hormone deficiency (GHD) in both children and adults concluded that incidence and prevalence of GHD are highly variable with findings from 1.2-33 per 100,000 persons per year to 4.6-40.6 persons, respectively [3]. It is estimated that over 50 000 adults in the United States are growth hormone deficient, with approximately 6 000 new cases reported yearly (this figure includes children with growth hormone deficiency that are transitioning to adulthood) [4].

In addition to physiologic symptoms, GHD for adults is also associated with impaired concentration and loss of memory, dissatisfaction with body image, and decreased quality of life [5]. Important areas of impact for AGHD include energy or vitality levels, mood, social isolation and self-control [6, 7]. Adults with AGHD may also experience psychological impairments such as depression, anxiety and social isolation [8–12].

Patient reported outcome (PRO) measurement for conditions such as AGHD where negative impacts are numerous is essential to assist clinicians, policy makers, payers and patients in understanding and deciding upon treatment options. Unfortunately, currently available measures are either not disease specific and have not been developed for or validated within the population of people with AGHD [13–15], do not have sufficient supporting documentation [16], or use yes/no response options that do not permit an assessment of degree of impact or smaller changes in treatment efficacy [17]. This qualitative study was conducted as the first step in the development process of a new PRO measure which will address the shortcomings of previous measures by assessing the full spectrum of impacts in the daily life of patients, and capture both small as well as larger effects of treatment on these impacts. Following FDA guidelines in the development of PROs, expert interviews with clinicians were conducted in combination with patient focus group interviews and literature review. This data was synthesized to support a conceptual model for the impact and consequences of AGHD. The purpose of this paper is to present the qualitative findings from this study.

## Methods

Seven semi-structured (open ended questions with follow up probes) focus groups and four telephone interviews were conducted with adults diagnosed with AGHD. They were held in three countries (Germany, United Kingdom, and United States) and six cities (Frankfurt, London, Munich, New York, Dallas, and San Francisco). Eligibility for participation included: 1) 22 years of age or older; 2) diagnosed with AGHD of any etiology; 3) diagnosed by a physician and able to provide proof of diagnosis with GH (growth hormone) medication prescription for GH medication, or letter from a physician stating diagnosis; and 4) both on and off treatment for AGHD at least once as an adult. The telephone interviews were conducted with residents of New York City (1) and London (3) in order to accommodate the participants’ inability to attend the focus group discussion. Utilizing purposive sampling, adults with AGHD were recruited through an international professional market research organization using their proprietary database of potential study participants. All participants gave their informed consent prior to their inclusion in the study and received an honorarium for their participation commensurate with their time and effort. This project’s ethics approval was granted by Independent Review Consulting, Inc. IRB (#09012-01).

The focus group and telephone interview guides were designed to elicit the burden of illness and treatment impacts of AGHD. The guides were comprised of open-ended questions concerning perceived impacts of AGHD on social, physical, and psychological aspects of life, productivity, treatment impacts and satisfaction, and compliance. They were developed from preliminary research through literature review, conducted using the US National Library of Medicine’s PubMed database, and interviews with six clinicians with AGHD expertise.

The focus group interviews were moderated by professional focus group leaders, and in the native language of the participants. They were conducted in professional market research focus group facilities. All of the English-language telephone interviews and focus group interviews were moderated by the first author, who is a licensed mental health clinician and trained group moderator. Professional facilitators moderated the focus group interviews conducted in Germany. To ensure consistency between groups, all facilitators were trained by the first author. The first author also observed all groups in person and listened to respondents via simultaneous translation. The focus group interviews were approximately two hours in length and the telephone interviews were one hour in length.

The focus groups and telephone interviews were audio taped, translated into English when conducted in a non-English language, and transcribed verbatim. They were analyzed (hand coded) and synthesized thematically using a multi-step process: (1) individual interview statements were examined; (2) statements were summarized; (3) statements were grouped and coded into categories; and (4) moderating variables (factors which may influence the strength of the relationship between Growth Hormone Deficiency (GHD) and impacts) were examined [18, 19].

Concurrent with conducting the focus groups, six clinical experts were identified by the first author as established clinical experts in the field (endocrinologists and nurse educators) who saw at minimum 100 AGHD patients yearly. The clinicians were interviewed individually by telephone for approximately 1 hour each. These clinicians identified key impacts of AGHD on patients: energy levels, personal well-being, and body mass. Furthermore, these clinicians acknowledged that existing measures designed to assess impacts of AGHD were not sensitive to the range and gradations of impact that AGHD poses for patients.

## Results

### Sample characteristics

The study included a total of 39 respondents (see Table 1), 23 (59%) females and 16 (41%) males. Participants resided in Germany (n = 14, 36%); the United Kingdom (n = 12, 31%), and the United States (n = 13, 33%). The average age was 50.7 years (range 22–82). A majority of respondents (n = 24, 62%) reported pituitary disease or pituitary tumor as the cause of their GHD; however, for some the etiology was unknown (n = 6, 15%). A majority (n = 34, 87%) was taking medication at the time of the focus groups. The average age for initiating treatment for GHD was 43 years (range 5–71). The average age of GHD diagnosis was 39.7 years (range 4–71). Sixteen (41%) were not working, although education levels were distributed across all categories.

The average number of self-reported comorbid conditions per patient was 2.8. Conditions reported include: endocrine disorders - including diabetes and thyroid disorders (n = 29, 74%); metabolic conditions - including elevated cholesterol (n = 16, 41%); arthritis, rheumatic diseases, musculoskeletal conditions (n = 13, 33%); mental health conditions - including depression and anxiety (n = 11, 28%); and heart disease and/or cardiovascular condition - including hypertension (n = 9, 23%). These comorbid conditions were unsurprising, representative of the health problems associated with AGHD in general.

#### Comparison across countries

The mean age of respondents was similar across countries, as was the mean age of AGHD diagnosis. However, there were relatively fewer respondents who were not employed in the United States (n = 4, 31%), as compared to Germany (n = 7, 50%) and the United Kingdom (n = 5, 42%). Respondents from the United Kingdom worked full time more frequently (n = 7, 58%) than did those from either Germany (n = 4, 29%) or the United States (n = 5, 38%). The primary cause of AGHD in Germany and the United Kingdom was pituitary disease/tumor (Germany: n = 12, 86%; United Kingdom: n = 8, 67%). However, in the United States, a range of causes existed for respondents, including pituitary disease/tumor (n = 4, 31%), head trauma (n = 2, 15%), and unknown etiology (n = 4, 31%). Nearly all respondents from Germany and the United Kingdom were taking GH treatment at the time of the focus group interview (Germany: n = 13, 93%; United Kingdom: n = 12, 100%). However, in the United States only 9 (69%) respondents were taking GH. The mean age the respondents initiated GH treatment was similar across countries.

**Table 1:** Sample characteristics

Sample size	Germany	United Kingdom	United States	Total
	(n = 14)	(n = 12)	(n = 13)	(n = 39)
**Gender; n (%)**				
Female	8 (57)	6 (50)	9 (69)	23 (59)
Male	6 (43)	6 (50)	4 (31)	16 (41)
**Ethnicity; n (%)**				
Caucasian/White	3 (21)	11 (92)	9 (69)	23 (59)
Latino	0 (0)	0 (0)	3 (23)	3 (8)
Asian	1 (7)	1 (8)	0 (0)	2 (5)
Mixed	1 (7)	0 (0)	0 (0)	1 (3)
Other	0 (0)	0 (0)	1 (8)	1 (3)
Decline to report	9 (64)	0 (0)	0 (0)	9 (23)
**Marital Status; n (%)**				
Married	8 (57)	7 (58)	7 (54)	22 (56)
Single	2 (14)	3 (25)	4 (31)	9 (23)
Partnered	2 (14)	1 (8)	2 (15)	5 (13)
Divorced	2 (14)	0 (0)	0 (0)	2 (5)
Widowed	0 (0)	1 (8)	0 (0)	1 (3)
**Age; mean (range)**	53.1	52.5	46.4	50.7
	(22–77)	(23–82)	(22–71)	(22–82)
**Highest level of education; n (%)**				
High school/technical (or lower)	5 (36)	4 (33)	5 (38)	14 (36)
College degree	7 (50)	3 (25)	6 (46)	16 (41)
Graduate (or higher)	2 (14)	5 (42)	2 (15)	9 (23)
**Employment status; n (%)**				
Not working for pay	7 (50)	5 (42)	4 (31)	16 (41)
Full time for pay	4 (29)	7 (58)	5 (38)	16 (41)
Part time for pay	2 (14)	0 (0)	4 (31)	6 (15)
Student	1 (7)	0 (0)	0 (0)	1 (3)
**Age (years) at GHD diagnosis; mean (range)**	42.0	38.9	38.0	39.7
	(8–63)	(4–71)	(10–61)	(4–71)
**Cause of AGHD; n (%)**				
Pituitary disease/tumor	12 (86)	8 (67)	4 (31)	24 (62)
Head trauma	0 (0)	0 (0)	2 (15)	2 (5)
Short stature	1 (7)	0 (0)	1 (8)	2 (5)
Hypopituitarism	0 (0)	1 (8)	1 (8)	2 (5)
Acromegaly; removal of pituitary gland	0 (0)	1 (8)	1 (8)	2 (5)
Brain tumor	0 (0)	1 (8)	0 (0)	1 (3)
Unknown	1 (7)	1 (8)	4 (31)	6 (15)
**Currently on GH treatment; n (%)**				
Yes	13 (93)	12 (100)	9 (69)	34 (87)
No	1 (7)	0 (0)	4 (31)	5 (13)
**Age (years) first took GH treatment; mean (range)**	45.7^a^	44.3	38.9^b^	43.0^c^
	(8–63)	(5–71)	(11–61)	(5–71)
**Number other prescription meds currently taking; mean (range)**	5.0^a^	3.9	3.4	4.1
	(1–15)	(1–7)	(0–7)	(0–15)
**Total AGHDA** ^**d**^ **score per country; mean (range)** ^**e**^	7.9	9.8	11.5	9.7
	(0–20)	(0–22)	(0–23)	(0–23)
**Number co-morbid conditions; mean (range)**	3.4	2.2	2.6	2.8
	(1–7)	(0–4)	(0–8)	(0–8)

^a^One blank response.

^b^One participant had never been on medication.

^c^Two missing responses, n = 37.

^d^AGHDA: Quality of Life in Adult Growth Hormone Deficiency Assessment.

^e^Scored 1 point for positive response, possible range 0–25.

### Themes generated by focus groups

The analysis identified five domains of impact: *Psychological Health, Physical Health, Cognition, Energy Loss,* and *Treatment Effects*. Total saturation of themes was reached after the sixth focus group. When considered by domain, saturation was reached after the second focus group for themes related to physical health and cognition, and by the third focus group for themes related to daily life impacts due to energy loss. Saturation was reached by the sixth focus group for themes related to psychological health and treatment effects.

### Psychological health

Psychological health themes were the most dominant themes discussed in these focus groups and interviews. Respondents struggled with body and self-image and reported a wide range of emotional impacts of AGHD, including frustration, anxiety, anger or resentment, depression, irritability, and mood swings. Respondents reported tension in relationships, and reduced social interaction as a result of AGHD.

Very generally, respondents linked their troubles with body image to the weight gain associated with AGHD, whereas they often linked their troubles with self-image to fatigue and low energy. Body image was discussed in five focus groups and two telephone interviews, and self-image was discussed in four focus groups and two telephone interviews:

#### Body image

*Oh, horrible. Just, I just think I look horrible and I feel really embarrassed when people see me who haven’t seen me since my illness. If I bump into people who I haven’t see for a while, I think they must be looking at me and thinking, oh my God, look how she’s let herself go. I do feel very embarrassed at how I look. I never feel that I look nice. (UK)*

*Yes, I somehow felt bad because I put on a lot of weight and my father measures 1,70 m and weighs 100 kg and then suddenly I felt that I look very much like him. I needed new clothes and everything. It was really horrible to look like that and I felt bad about it. (Germany)*

#### Self-image

*It was difficult. And I would complain to my wife, not a lot of other people. She could tell that I was not my same self. (USA)*

*You feel a bit of a failure that you’re not there really to give the support that you always had given. And also you hadn’t got the energy, I always used to sit every night with them and do reading and homework and things like that and I suppose that went so yeah, I suppose it did affect me in that way, I did feel a bit of a failure, I suppose in that respect. (UK)*

Depression was a common theme, and respondents spoke about depression and AGHD in five focus groups and three telephone interviews:

*I was depressed all the time, and the doctor had me on antidepressants for a good three years, and then finally I managed to get him to agree to send me back to the hospital. (UK)*

Furthermore, respondents spoke about feeling easily stressed and anxious:

*Anxiety, like right now; there are times when I just sit down and I can just feel like sometimes my whole insides are just shaking and I have to take these deep breaths. (USA)*

Additional emotional impacts of AGHD included lack of motivation, frustration, anger, irritability, and mood swings. In combination, the psychological impacts resulted in consequences for respondents’ relationships. Many described social tensions with others, either as a direct result of the impacts of AGHD or as something that is difficult to talk about with friends, and these themes were discussed in all seven focus groups and three telephone interviews:

*The latter years it ruined my marriage, and my ex husband feels it’s what made him an alcoholic because of the trauma of the stuff I went through. (UK)*

*For example, on the weekend we're going to do that – and then I feel bad and I'm tired and I can't do that. And that creates problems. Of course, he doesn’t tell me but then he is disappointed. Again, we can't do that! You said you'll meet with those friends and then you can't go there. And I try to do it, really, I try but I feel bad. (Germany)*

*It's like invisible for us, but at the same time it's kind of like a detriment that people don't know that you have it because they see you and you look fine and you look fine and then you feel like hell. So people look at you like, "Well why are you fatigued?" or "Why do you not feel like you can finish this project?" that's stressful. It's like too much effort to even tell somebody what's going on with you and explain it. "You're obviously tall. You haven't suffered as a result of this." … It's not physical to them. If it's not physical to them, it's not valid to them. (USA)*

In summary, respondents reported that the psychological impacts of AGHD were substantial, and extended to how they perceive their bodies and themselves, as well as how they are able to interact with family and friends.

### Physical health

Respondents attributed a variety of physical health impacts to AGHD, including sleep troubles, fatigue, loss of sex drive, weight gain, hair and skin issues, muscle and bone loss, and metabolism/digestion problems.

Of these, the most frequently cited were sleep troubles, and fatigue. Respondents had difficulty falling asleep or sleeping through the night, and were deeply fatigued during the day. This resulted in additional daytime sleeping, sometimes inappropriately (such as in the middle of a task or conversation). These themes were noted in all seven focus groups and three telephone interviews:

#### Sleep

*But it was the sleep really, well, my sleep patterns were completely mixed up and just the tiredness all the time. (UK)*

*I really have a bad sleep. First of all, I fall asleep really late. In most cases, it's eleven or midnight although I go to bed at nine knowing I have to go to school the next morning and it takes forever to go to sleep. And then I wake up again after two hours and then I sleep again and then it continues like that. (Germany)*

#### Fatigue

*Oh, it’s really terrible. When I was at my lowest I spent most of the day either sleeping or lying on the living room floor with my feet on the couch. I was just so tired I could not even get up and do anything. (USA)*

*I was so tired. I didn’t take pleasure in anything. (Germany)*

Respondents also reported loss of sex drive and desire as an impact of AGHD. This theme was discussed in all seven focus groups and two telephone interviews:

*There is zero sex drive whatsoever. […]No desire. I can look at my girlfriend and she’s a really adorable, cute, sweet, beautiful person and I’m taken aback by her, but there is not the impetus to go any further. It just takes a lot of energy to cross that bridge. (USA)*

*The mood is different. Let me put it like this. I mean as everybody says if you're tired, exhausted and worn out, then, of course, you wouldn’t want any sexuality. (Germany)*

Additionally, respondents noted weight gain due to AGHD as an impact that was occasionally misunderstood early in the diagnostic process. A few respondents reported muscle mass and bone losses, metabolism/digestion problems, and high cholesterol associated with AGHD.

### Cognition

Respondents reported that problems with memory and an ability to focus or concentrate were associated with AGHD. They also reported a general feeling of being slower than others. These themes were discussed in all seven focus groups and three telephone interviews:

#### Focus or concentration

*My concentration is quite bad. I used to love studying and reading but now it, it’s a struggle for me just to read like a paragraph in a magazine article, I just find that I can’t concentrate long enough. (UK)*

*I start talking and it goes. That’s the thing: it’s hard to hold onto something you know you’re about to say. (USA)*

#### Memory problems

*I feel, I forget things. People sometimes say do you remember when we did this and that? And I say, yes but I can't remember things from my past. And things that people tell me now, I forget it in a few moments. (Germany)*

*I very nearly lost my job because I was forgetting to do things. When you know you’ve got a bad memory you can write lists and you can set, particularly with electronic calendars, and now I have things pop up even to remind me to take my mobile home with me. […]But it was, that was, I was tired but I wasn’t sure whether it was just the fatigue that made me forget things or whether I generally forgot things and now I know that it’s I generally can’t remember things. (UK)*

Respondents were bothered by their memory loss and inability to focus, and described these impacts as substantial in their lives, creating self-doubt in their abilities.

### Energy loss

Energy loss was implicated in all of the major impacts to daily life, including work, accomplishing home tasks such as cleaning or cooking, exercise or sports, and productivity. Respondents reported that low energy and profound fatigue made it difficult to do the activities they had accomplished easily prior to acquiring AGHD. Many respondents reduced their hours of paid work or changed their jobs to accommodate their loss of energy, and some actually stopped working for pay because it proved to be so difficult. Work losses were described in every focus group and three telephone interviews:

*Well, I used to work 40 hours a week. I can’t do that anymore. I work maybe 15, 20 hours, you know, because I’m always tired. I wake up tired and just can’t do much. (USA)*

*I’ve had to give up my job. […] I did try to go back to work last year for, I tried for about six months, for fifteen hours a week, but it just didn’t work out, I just hadn’t got the mental capacity or the physical capacity really to do it, to take the stress, the speed that you have to work and the accuracy, I just couldn’t cope with it any more. (UK)*

At home, respondents were unable to shop, cook, or clean and often relinquished these tasks to others in the household. If they were able to accomplish the task, it was often through a great deal of effort and with breaks from the task. These experiences were noted in six focus groups and three telephone interviews:

*I was in a very bad condition. After work, having to do the household [chores]. And I didn’t feel like doing anything. I was just tired and exhausted. Really worn out. (Germany)*

*My mum would have to do the shopping for me, she’d come round, around lunchtime, she’d perhaps make me some lunch. I’d be asleep again when they came home from school and work, so they’d be waking me up when they came in. Nothing would be prepared for them to eat. I wouldn’t have achieved anything during the day, and just slept most of the day. (UK)*

Several individuals recalled the time prior to AGHD when they were active physically, participating in aerobic activities such as biking or running for exercise and playing sports. They were no longer able to do these activities once they had acquired AGHD. This theme was reported in five focus groups and three telephone interviews:

*[Compared with prior to being GHD] I would ride my bike to work three or four times a week. It’s a four-mile bike ride. That’s where I got my joy was walking and riding my bike. I don’t do it anymore. I did yoga on a regular basis. I didn’t do it [anymore]. (USA)*

*I love, for example, to do sports but I couldn’t do it anymore. (Germany)*

Generally, respondents felt that accomplishing any task was challenging and they felt this as a loss of productivity both at work and at home. Loss of productivity was noted in six focus groups and four telephone interviews:

*I think that just feeling of not being able to do anything, of just not being able to even walk to the end of the street and back, not feeling like I could participate in anything because of it. (UK)*

*I want to become productive again and I want to feel better. (Germany)*

*I’d no energy to do anything at all, nothing whatsoever. (UK)*

In summary, energy loss due to AGHD sustained substantial impacts in daily life for these respondents, particularly in the areas of work and home life.

### Treatment effects

Treatment with growth hormone positively impacted each of these domains for these respondents. Respondents reported that while on treatment, they lost weight and had more energy and motivation for exercise. This, in turn, improved their body image. They reported feeling better. As a result, their self-image improved and they became more socially active. Respondents stated that they had an improved ability to cope with the anxieties and stresses of life, and had fewer emotional swings or less depression. In general, they were more motivated to return to the activities that they had enjoyed previously, before they acquired AGHD.

Physically, they slept better and felt less fatigue during the day and experienced more energy. Some gained back muscle tone and felt stronger, although the loss of sex drive reported by respondents was improved for only two people. Hair growth returned for some respondents. However, respondents reported that treatment did not seem to improve problems with memory loss and focus or concentration very substantially.

Importantly, with treatment, respondents were able to resume their hobbies and exercise activities, and reported that they were able to accomplish their household tasks again. For those who were working, their work lives and productivity had improved, and a few who had not been working were considering a return to work because they felt better and had more energy with treatment.

*I was happy to get the product because I could more actively participate in life again. And with much more fun and pleasure and being able to participate in sports exercises again. Many things I didn’t do in the past. (Germany)*

*Also, with the growth hormone you could accomplish certain things that you wouldn’t be able to do. You can’t understand it because you have energy. (USA)*

*I feel good actually, very good at the minute, previously I felt like I was, had a life of a 90 year old lady and the energy levels of a 90 year old lady and now I feel like I’m probably about 25, but I’m not. But, yeah, no, I certainly have more energy and just, I just feel good. (UK)*

### Theoretical framework of the impact of AGHD

**Figure 1:**
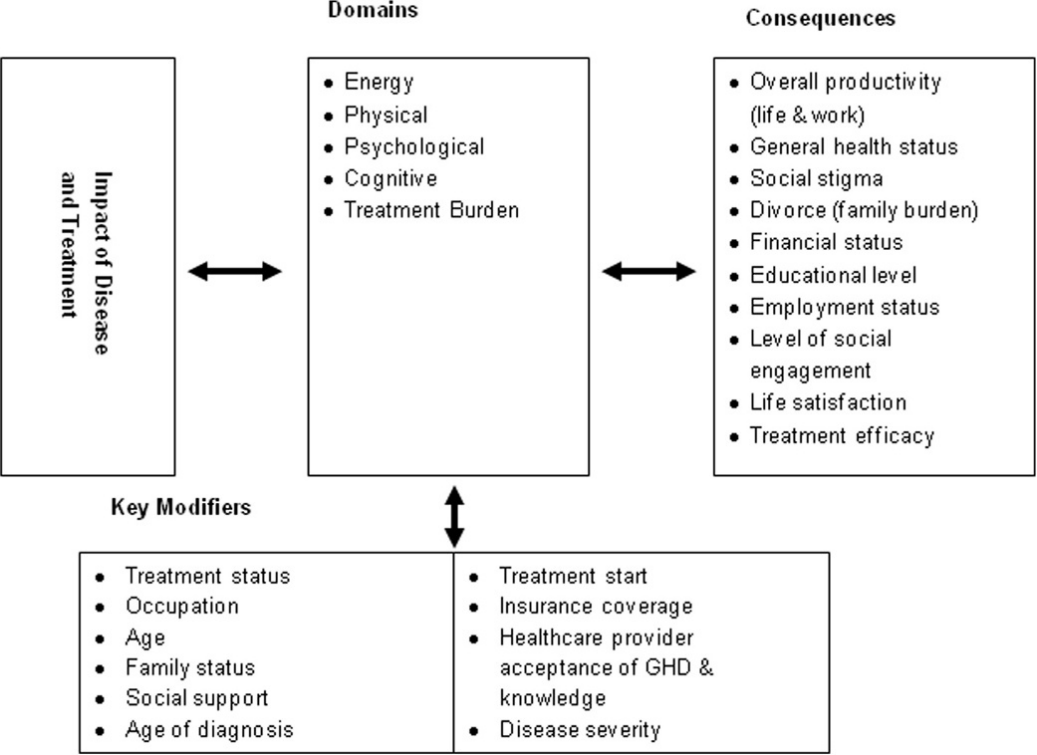
**Theoretical framework for impacts of AGHD.**

From these data, a theoretical framework was developed to illustrate the domains of AGHD impact, their consequences, and factors that influenced individual experience. As shown in the model, the main domains the GHD impacts for adults are Energy, Physical, Psychological, Cognitive and Treatment Burden. Additionally, there are modifiers which can influence, either ameliorate or intensify, the degree that disease impacts the person (see Figure 1).

## Discussion

This study demonstrates that the burden of illness for AGHD is substantial, with impacts to both psychological and physical health related quality of life, including the essential aspects of daily life such as work, home activities, and social relationships. As also shown in the theoretical framework, these impacts are influenced by multiple factors, some of which can be intervened with while others cannot. It is important from both a clinical and research perspective to understand and assess these modifiers, especially the ones which can be influenced, such as level of social support. Clinicians may then be able to include recommendations for additional treatments such as therapy or support groups when needed. Further, researchers should be aware that these variables may be important covariates when analyzing data from studies including a measure of impact. Additionally, understanding the longer term impacts of GHD on a person’s daily life and functioning will allow clinicians to incorporate discussion of these consequences with their patients, which may lead to a more realistic understanding of their disease for both the clinician and the patient. Of importance is that the direction of the influences between domains, consequences, and modifiers is bi-directional as supported by our findings that treatment for AGHD may alleviate many of the difficult impacts associated with AGHD.

Importantly, as demonstrated with the data, there are strong consequences for untreated AGHD, including overall life and work productivity; health; social stigma; partnership disruption such as difficulty or divorce; impacts on employment; educational attainment and ultimately financial status; social engagement; and life satisfaction.

This study contributes a strong international focus to any discussion on the impacts of AGHD, with compelling evidence that not only are the impacts substantial, but relevant to people with AGHD in both the United States and Europe. Respondents were in strong agreement across countries about the impact of AGHD on psychological, physical, cognitive, and energy aspects of life. They expressed similar concerns about the effects of these impacts on work, home, and social relationships, suggesting that the major driver responsible for the impacts of AGHD is the disease and not culturally specific attitudes or beliefs.

A small sample size of 39 participants is a limitation of this study. Recruitment was based on a database comprised of voluntary patients, and therefore, the study sample may not be representative of all individuals with AGHD. Additionally, allowing participants with AGHD of any etiology and the lack of information regarding all other concomitant hormonal dysfunctions creates obstacles to a close-grained analysis delineated by hormonal status and treatments for other hormonal dysfunctions. The extent to which the impacts reported in these focus groups was related to the loss of the pituitary gland and related treatments instead of the GH deficiency itself was not always clearly detailed in these focus groups, or by these respondents. However, since many of the respondents had multiple hormonal dysfunctions, the impacts of these dysfunctions may have influenced some of their responses.

Future research should continue to develop an understanding of impacts by etiology of AGHD, through both qualitative and quantitative methodologies. As noted earlier, the expert clinicians interviewed during this study were dissatisfied with current measures available for assessing impacts of AGHD on patients. Additionally, the existing measure is not sensitive to change in symptom or impacts, and other adapted measures have not been developed for or validated within the population of people with AGHD. The development of newer measures, which capture these multiple impacts and allows for a wider variation in severity response options, may help to address this gap in assessment of AGHD as well as tailor treatment plans for individual patient’s circumstances.

## Conclusions

The respondent data from this study demonstrates how important it is to be able to assess patient impacts fully, and with an attention to the range and severity that patients actually experience. A new measure with more sensitivity than the existing measures would be helpful to clinicians and regulatory agencies to assess the benefits of treatment.
